# Nuclear Imaging to Detect Diaphragmatic Perforation as a Rare Complication of Microwave Ablation

**DOI:** 10.1155/2017/6541054

**Published:** 2017-03-14

**Authors:** Stephanie Cull, Gebran Khneizer, Abhishek Krishna, Razi Muzaffar, Sameer Gadani, Zafar Jamkhana

**Affiliations:** ^1^Department of Internal Medicine, Saint Louis University School of Medicine, St. Louis, MO, USA; ^2^Division of Pulmonary, Critical Care and Sleep Medicine, Saint Louis University School of Medicine, St. Louis, MO, USA; ^3^Radiology Department, Saint Louis University, St. Louis, MO, USA

## Abstract

Acquired diaphragmatic perforation leading to massive hepatic hydrothorax and respiratory failure is a rare complication of microwave ablation (MWA) of hepatocellular carcinoma (HCC). Imaging modalities to detect pleuroperitoneal communication remain poorly described. We report a nuclear imaging technique used to efficiently diagnose and locate diaphragmatic defects. A 57-year-old male with cirrhosis and HCC presented with respiratory distress after undergoing MWA of a HCC lesion. He was admitted to the intensive care unit for noninvasive positive pressure ventilator support. Chest radiography revealed a new large right pleural effusion. Large-volume thoracentesis was consistent with hepatic hydrothorax. The fluid reaccumulated within 24 hours; therefore an acquired diaphragmatic perforation induced by the ablation procedure was suspected. To investigate,  ^99m^Technetium-labeled albumin was injected into the peritoneal cavity. The tracer accumulated in the right hemi thorax almost immediately. The patient then underwent transjugular intrahepatic portosystemic shunting in efforts to relieve portal hypertension and decrease ascites volume. Unfortunately, the patient deteriorated and expired after few days. Although diaphragmatic defects develop in cirrhotic patients, such small fenestrations do not normally lead to rapid development of life-threatening pleural effusion. MWA procedures can cause large diaphragmatic defects. Immediate detection of this complication is essential for initiating early intervention.

## 1. Introduction

Hepatic hydrothorax is defined as a pleural effusion volume greater than 500 mL in a patient with liver cirrhosis, not otherwise explained by a cardiopulmonary or primary pleural process [1–3]. Only 1-2% of pleural effusions are secondary to cirrhosis and only 5–10% of patients with cirrhosis are affected by hepatic hydrothorax [3]. The exact pathogenesis of such effusions remains poorly understood. Many agree upon the theory that ascitic fluid passes directly through diaphragmatic defects (either congenital or acquired), which constitute direct pleuroperitoneal communications in the form of holes or blebs [3–5]. The condition may even occur in patients with minimal ascites [6]. Negative intrathoracic pressure draws ascitic fluid directly into the pleural space, more commonly to the right thorax considering the right hemidiaphragm is thinner and less muscular than the left counterpart. These pleuroperitoneal communications are often small fenestrations through which fluid slowly accumulates in the thorax. However, literature describes cases of large diaphragmatic defects which lead to accumulation of massive fluid volume in the pleural space with subsequent respiratory failure, often requiring daily thoracentesis or chest tube placement [4, 6]. Chest tube placement is relatively contraindicated in hepatic hydrothorax as it leads to rapid fluid depletion and electrolyte imbalance [3]. Although this patient population may benefit from transjugular intrahepatic portosystemic shunting (TIPS), there are many cases where the patient cannot tolerate this procedure due to rapid clinical decompensation.

Acquired diaphragmatic defect is a rare complication of microwave ablation (MWA), a form of thermal ablation treatment for cancer, commonly hepatocellular carcinoma (HCC) [7, 8]. If the tumor is located on segment seven or eight in close proximity to the diaphragm, the heat deployed during ablation may lead to thinning of the diaphragm and widening of preexisting fenestrations or perforation of blebs [9]. Despite the ability to discover diaphragmatic defects through thoracoscopy and even autopsy [5], imaging techniques to visualize these defects remain uncommon and not well described. We report a simple and efficient nuclear imaging technique to diagnose significant diaphragmatic defects.

## 2. Case Presentation

**Figure 1:**
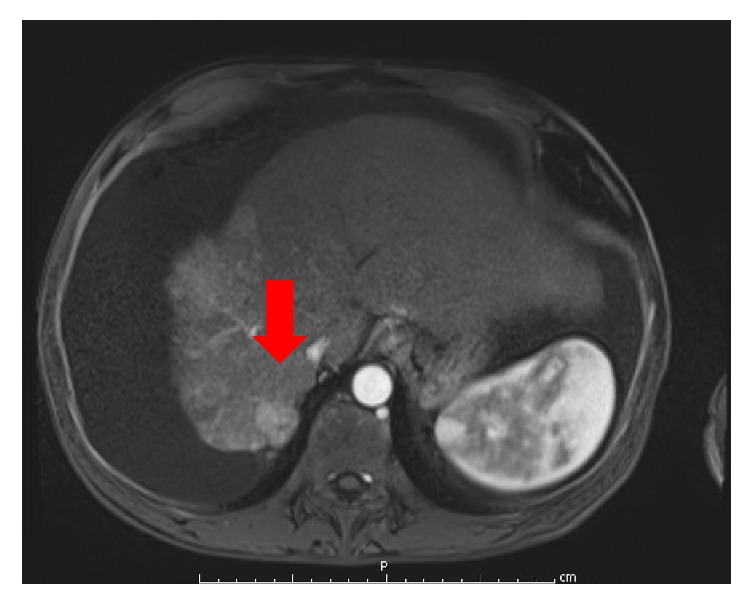
MRI image of an arterially enhanced lesion representing hepatocellular carcinoma seen with washout and delayed capsular enhancement. The tumor measures 2.2 cm in diameter and is located on segment 7 of the liver in close proximity to the tendinous portion of the right hemidiaphragm.

A 57-year-old male with a past medical history of cirrhosis and HCC secondary to chronic alcohol abuse and hepatitis C infection was undergoing serial transarterial chemoembolization (TACE) procedures. He also had recurrent ascites refractory to both low sodium diet restriction and diuretic therapy. Prior to admission and in addition to TACE, the patient underwent MWA for an enlarging HCC lesion located on segment 7 of the liver (see Figure 1). A 20 cm probe (with a 3-4 cm ablation zone) was advanced under CT guidance and deployed across the lesion. After confirming the position of the probe, ablation was performed per protocol (65 watts for 10 minutes). An overlapping session was also performed. Immediately after the MWA procedure, the patient underwent therapeutic large-volume paracentesis (8 L), without complication, and was discharged home afterwards.

**Figure 2:**
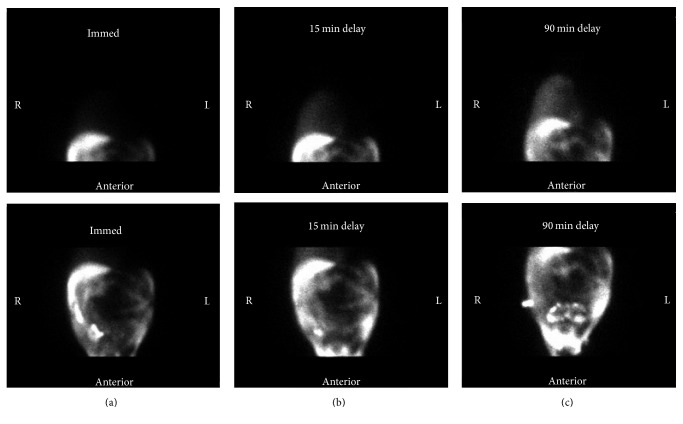
Anterior planar nuclear images of the thorax (upper images) and abdomen (lower images) obtained after radiotracer administration with ^99m^Technetium-labeled albumin into the peritoneal cavity. Images were obtained immediately after tracer injection (a) and at intervals of 15 minutes (b) and 90 minutes (c). Very faint radiotracer accumulation is evident in the right hemi thorax immediately (a) and is more pronounced at 15 minutes (b) and at 90 minutes (c). Radiotracer is seen to equalize in the abdomen over time (bottom images of (a), (b), and (c)). There is no visible tracer in the left hemi thorax.

Three days after procedure, the patient presented to the emergency department with respiratory distress reporting progressive shortness of breath which began the day after MWA. Vital signs were significant for a respiratory rate of 33 breaths per minute and oxygen saturation of 87% while breathing ambient air. Physical exam revealed a cachectic-appearing man in respiratory distress using accessory muscles of respiration. There were diminished breath sounds on auscultation of the right lung fields. The patient was admitted directly to the intensive care unit for noninvasive positive pressure ventilation support. Chest radiography showed a large right-sided pleural effusion. Computed tomography of the chest confirmed the effusion and ruled out pulmonary embolism and lipiodol embolism. The patient had no prior history of pleural effusion or evidence of such on previous imaging studies. Thoracentesis was performed and pleural fluid analysis revealed a transudative fluid consistent with hepatic hydrothorax. Pleural fluid microbiology was negative for infection and cytology studies were negative for malignancy. The effusion reaccumulated within 24 hours with associated atelectasis of the right lung. A bedside ultrasound revealed minimal ascitic fluid, even though the patient was known to have significant refractory ascites. With careful consideration of the clinical timeline, an acquired diaphragmatic perforation induced by MWA therapy was suspected. Therefore, a nuclear medicine peritoneal shunt study was performed whereby 4.0 mCi Technetium-99m mixed with albumin was injected into the peritoneal cavity under sterile conditions. Planar nuclear images of the chest and abdomen were obtained immediately and at intervals of 15 minutes and 90 minutes after radiotracer administration (see Figure 2). Tracer accumulation in the right hemi thorax was evident on the image taken immediately after injection (see Figure 2(a)). The patient later required frequent thoracenteses and therefore a chest tube was necessary (weighing the benefits against the known risks associated with placing a chest tube). The decision was then taken to pursue TIPS in an effort to relieve portal hypertension and decrease the volume of ascitic fluid formation. Intubation was required for the procedure with subsequent inability to extubate afterwards. The patient's clinical status declined further as he developed atrial fibrillation and distributive shock. Upon discussion with the family and thoracic surgery service, together it was decided to not pursue a surgical evaluation of the diaphragmatic perforation. Furthermore, the decision was made to pursue comfort care measures as per the patient's previously stated wishes to the family. The patient passed away.

## 3. Discussion

MWA has become widely utilized as a form of cancer treatment in multiple medical disciplines with the advances in interventional technology. Recently, such modules became an alternative treatment for liver malignancy, particularly for those who are poor surgical candidates. Postprocedure complications are uncommon and include hemorrhage, bile leak, fluid collection/abscess formation, cholangitis, tumor seeding, and abdominal pain [10].

Acquired diaphragmatic perforation has been reported as a rare complication of MWA for the treatment of HCC [8, 9]. Although diaphragmatic defects are seen in patients with cirrhosis who develop hepatic hydrothorax, they are usually described as small fenestrations that do not lead to rapid development of pleural effusion [5]. It is hypothesized that the heat generated from the ablation procedure stretches the small fenestrations or leads to perforation of preexisting blebs of the diaphragm [9]. Another potential explanation for such large defects is the microwave needle penetrating the diaphragm. As a result, large amount of ascitic fluid is drawn into the pleural space secondary to the negative intrathoracic pressure.

Even though multiple imaging modalities have been reported for indirect measurement of pleuroperitoneal communication across the diaphragm, such imaging techniques remain uncommon and not well reported. First, an indirect visualization of colored dyes such as methylene blue and indocyanine green was injected into the peritoneal space and migrated into the pleural space [4, 11]. Then MRI has been used to visualize a hypointense jet across the diaphragm in both T1- and T2-weighted sagittal scans [11]. But this modality is both time consuming and heavily dependent on patient participation. Furthermore, contrast-enhanced ultrasound detects such diaphragmatic defects by injecting the peritoneum with perflubutane microbubbles, an imaging agent that is safely eliminated from the lung. The patient then undergoes postural changes which elicits a jet-like flow of tracer-enhanced ascitic fluid into the hemithorax [8, 12]. These postural changes may not be clinically practical in critically ill patients. Another example is injecting radioisotope (such as ^99m^Tc-sulfur colloid) into the peritoneum and then visualizing the tracer in the hemithorax [13, 14].

The imaging modality that we employed offers several unique advantages in critically ill patients. First, it is time efficient and inexpensive. Second, it requires minimal patient participation and thus it can be performed on critically ill patients in contrast to previously described modalities such as MRI and contrast-enhanced ultrasound. Moreover, the low cost of such modalities is also promising. But availability of the nuclear agent, namely, ^99m^Technetium mixed with albumin, is the imaging technique's main limitation.

Identifying the diaphragmatic perforations early in the clinical course is essential to evaluate life-saving intervention and potential repair. Repair would include procedures such as chemical pleurodesis [6, 11] or video-assisted thoracoscopic surgery (VATS) [8]. Matono et al. described the clinical outcome of a patient who remained free of pleural effusion for over a year after closure of the diaphragmatic defect with sutures via VATS [8]. Liang et al. used a Dacron heart patch trimmed to appropriate size to close the lesion with minimal pleural effusion afterwards [9]. Such potential repair techniques offer a promising future for patient population with large diaphragmatic defects.

In conclusion, following MWA if a patient develops respiratory distress and a significant pleural effusion, early detection of diaphragmatic perforation using a nuclear peritoneal shunt study can be life-saving. This imaging is possibly the essential step between diagnosis and immediate intervention to avoid fluid and electrolyte depletion and respiratory clinical deterioration.
